# The Anti-Inflammatory Effect of the β1-Adrenergic Receptor Antagonist Metoprolol on High Glucose Treated Human Microvascular Retinal Endothelial Cells

**DOI:** 10.3390/cells11010051

**Published:** 2021-12-24

**Authors:** Giovanni Giurdanella, Anna Longo, Alfio Distefano, Melania Olivieri, Martina Cristaldi, Alessia Cosentino, Aleksandra Agafonova, Nunzia Caporarello, Gabriella Lupo, Carmelina Daniela Anfuso

**Affiliations:** 1 Biochemistry Section, Department of Biomedical and Biotechnological Sciences, School of Medicine, University of Catania, 95123 Catania, Italy; g.giurdanella@unict.it (G.G.); longo.anna@hotmail.it (A.L.); distalfio@gmail.com (A.D.); alessia1993@hotmail.it (A.C.); aleksandraaagafonova@gmail.com (A.A.); daniela.anfuso@unict.it (C.D.A.); 2 U.O. Clinical Pathology, Department of Hematology, AUSL Romagna, 47522 Cesena, Italy; melania.olivieri@auslromagna.it; 3 Fidia Farmaceutici S.p.A., 95123 Catania, Italy; mcristaldi@fidiapharma.it; 4 Department of Physiology & Biomedical Engineering, Mayo Clinic, Rochester, MN 55905, USA; Caporarello.Nunzia@mayo.edu

**Keywords:** diabetic retinopathy (DR), human retinal endothelial cells (HREC), β-Adrenergic Receptor (β-AR), metoprolol, Phospholipase A_2_ (PLA_2_), Kelch-like ECH-associated protein 1 (Keap1), nuclear factor erythroid-2-related factor 2 (Nrf2)

## Abstract

Hyperglycemia-induced impairment of the blood-retinal barrier represents the main pathological event in diabetic retinopathy that is elicited by a reduced cellular response to an accumulation of reactive oxygen species (ROS) and increased inflammation. The purpose of the study was to evaluate whether the selective β1-adrenoreceptor (β1-AR) antagonist metoprolol could modulate the inflammatory response to hyperglycemic conditions. For this purpose, human retinal endothelial cells (HREC) were treated with normal (5 mM) or high glucose (25 mM, HG) in the presence of metoprolol (10 μM), epinephrine (1 μM), or both compounds. Metoprolol prevented both the HG-induced reduction of cell viability (MTT assays) and the modulation of the angiogenic potential of HREC (tube formation assays) reducing the TNF-α, IL-1β, and VEGF mRNA levels (qRT-PCR). Moreover, metoprolol prevented the increase in phospho-ERK1/2, phospho-cPLA_2_, COX2, and protein levels (Western blot) as well as counteracting the translocation of ERK1/2 and cPLA_2_ (high-content screening). Metoprolol reduced ROS accumulation in HG-stimulated HREC by activating the anti-oxidative cellular response mediated by the Keap1/Nrf2/HO-1 pathway. In conclusion, metoprolol exerted a dual effect on HG-stimulated HREC, decreasing the activation of the pro-inflammatory ERK1/2/cPLA_2_/COX2 axis, and counteracting ROS accumulation by activating the Keap1/Nrf2/HO-1 pathway.

## 1. Introduction

Diabetic retinopathy (DR), a major cause of blindness, interrupts the physiological interaction between the vascular and neural components of the retina, causing vascular permeability, neovascularization, loss of the blood–retinal barrier (BRB) and, consequently, the loss of proper visual function [1]. A meta-analysis that analyzed published and unpublished population-based data for the causes of vision impairment and blindness between 1990 and 2020 reported an increasing prevalence of DR [2].

In the initial phase, defined as non-proliferative, DR is characterized by an increase in vascular permeability and by the onset of microaneurysms and hemorrhage. At the most advanced stage, DR is characterized by neovascularization and is called proliferative DR. During this phase, new, abnormal blood vessels are created [3] and patients have severe vision impairment [4]. The diagnosis of DR is based on the detection of microvascular lesions that are related to ocular angiogenesis. Some patients benefit from anti-vascular endothelial growth factor (VEGF) therapy, however, unfortunately, most patients fail to achieve clinically meaningful visual improvement and DR treatment remains challenging [4,5]. Therefore, there is an urgent need to develop new treatments.

Much of the literature has highlighted the controversial role of β-adrenoreceptor (β-AR) in the reduction or increase of the vascular impairment that is induced by hyperglycemia [6]. β-ARs are widely expressed in both the central and peripheral nervous system and mediate important functions following activation by catecholamines, such as bronchodilation, vasorelaxation, heart rate regulation, and neurotransmitter release [6]. Increased plasma levels of catecholamines have been observed in the pre-retinopathic stage and become highly significant in patients with early- or late-stage DR, suggesting their important role in DR pathogenesis [7]. Data that were reported by Sorriento et al. showed that endothelial cells express all the enzymes that are involved in the synthesis of catecholamines in response to hypoxia [8].

In vitro data showed that β1-AR stimulation reduced inducible nitric oxide synthase (iNOS) expression and modulated other hyperglycemia-induced inflammatory molecules, such as interleukin (IL)-1β, tumor necrosis factor-α (TNF-α), and prostaglandin E2 (PGE_2_) in human retinal endothelial cells and rat Müller cells through the involvement of PKA, p38 MAPK, and p42/p44 MAPK [9,10].

The correlation between DR and inflammation, in both type 1 and type 2 diabetes, highlights the possibility of alternative therapies for targeting inflammatory processes [11]. Phospholipases A_2_ (PLA_2_) catalyzes the hydrolysis of the *sn*-2 position of membrane glycerophospholipids, mostly phosphatidylcholine, to release arachidonic acid (AA), a precursor of eicosanoids including prostaglandins (PGs) and leukotrienes (LTs) [12]. Mammalian tissues contain three groups of PLA_2_, namely, cytosolic (cPLA_2_), secretory (sPLA_2_), and Ca^2+^-independent PLA_2_s, that are characterized by enzymatic properties and structures. The role of cPLA_2_ in diabetic retinopathy has been demonstrated. In particular, in an in vitro model of BRB, high or fluctuating glucose levels were effective in inducing cPLA_2_ activity as well as PGE_2_ and VEGF release in retinal endothelial cells in co-culture with pericytes. These effects were reversed after transfecting EC with small interfering RNA that was targeted to PLA_2_ [13]. The activation of cPLA_2_ responds to various pro-inflammatory stimuli that trigger a Ca^2+^-dependent mechanism of translocation from the cytosol to perinuclear membranes and the phosphorylation of its Ser-505 in the catalytic domain by the mitogen-activated protein kinase (MAPK) ERK1/2 and its Ser-727 by other MAPKs [14,15].

Reactive oxygen species (ROS) accumulation is a pivotal factor in hyperglycemia-induced retinal endothelial cell dysfunction, stimulating apoptosis and inflammation processes [16,17]. Cellular redox balance is maintained by conserved protective antioxidant and phase II detoxification responses in mammals that are regulated by the nuclear factor erythroid 2-related factor 2 (Nrf2), a transcription factor that is encoded by Nfe2l2 gene [18,19]. Nrf2 activation induces the expression of several genes, including heme oxygenase-1 (HMOX1), that contains antioxidant response elements (AREs) in its regulatory elements [20]. Kelch-like ECH-associated protein 1 (Keap1) is the main regulator of Nrf2 activity by keeping it in the cytoplasm and thereby inducing Nrf2 to proteasomal degradation [21]. High levels of glucose were shown to trigger Nrf2/ARE pathway inhibition and the consequent oxidative injury in endothelial cells [22]. It was observed that the cellular response against oxidative damage promoted the nucleus enrichment of Nrf2 and the concomitant decrease in Keap1 levels, placing new emphasis on the study of new molecular strategies that mediate its activation [22,23].

In the present study, we examined the effects of the β-AR antagonist metoprolol and the β-AR agonist epinephrine on human retinal endothelial cells (HREC) that were treated with a high glucose concentration in a model mimicking the processes that occur in proliferative retinopathy phases. We demonstrated that metoprolol was able to attenuate HREC dysfunction, ROS production, ERK ½-cPLA_2_ signaling activation, pro-inflammatory cytokines, and VEGF release.

Since β-ARs have been shown to modulate retinal dysfunction, understanding the cellular signaling that is involved in their modulation is critical for optimal drug development.

## 2. Materials and Methods

### 2.1. Reagents

Metoprolol (catalog n. ab120711) was purchased by Abcam (Cambridge, UK). Epinephrine (catalog n. E4375) was from Sigma Aldrich Co. (St. Louis, MO, USA). Rabbit polyclonal antibody against phospho-p44/42 MAPK (p-ERK1/2, catalog n. 9101), p44/42 MAPK (ERK1/2, catalog n. 9102), and GAPDH (catalog n. 2118) were purchased from Cell Signaling Technology (Danvers, MA, USA); mouse monoclonal antibody against Nrf2 (catalog n. ab137550) and mouse monoclonal antibody against TNF-α (catalog n. ab1793) were purchased from Abcam (Cambridge, UK). All the reagents that were used for cell cultures and stripping buffer were purchased from Invitrogen Thermo Fisher Scientific (Monza, Italy).

### 2.2. HREC Culture and Treatments

Primary human retinal endothelial cells (HREC) were purchased from Innoprot (Elexalde Derio, Spain) and were cultured with endothelial cell medium ECM that was supplemented with 5% fetal bovine serum (FBS), 1% endothelial cell growth supplement (ECGS), 100 U/mL penicillin, and 100 µg/mL streptomycin that were purchased from Innoprot. HREC were previously tested for their positivity for the von Willebrand factor by immunostaining [24]. Before cell seeding, the flasks or dishes were pre-coated with a bovine plasma fibronectin at a concentration of 1 mg/mL (Innoprot) for 1 h at 37 °C in a humidified atmosphere of 5% CO_2_ and were subsequently rinsed twice with sterile water. Before the treatments, HREC were incubated with medium containing 2.5% FBS that was supplemented ECM for 4 h before treatments. The cells were then treated with 5 mM glucose (normal glucose or NG, control condition) or with 25 mM glucose (high glucose, HG) in 2.5% FBS ECM medium for 24–48 h in the absence or in the presence of 1 and 10 μM of metoprolol and/or 1 and 10 μM of epinephrine. The cells were treated after reaching about 70% confluence and were used at passages 3 and 9. After treatment, the cells were washed twice in PBS and subjected to subsequent analyses.

### 2.3. Cell Viability

Cell viability was determined by the 3-[4,5-dimethylthiazol-2-yl]-2,5-diphenyl tetrasodium bromide (MTT assay, Chemicon, Temecula, CA, USA) as previously described [25]. HREC were seeded at 1.5 × 10^4^ cells per well into 96-well plates. After overnight growth, the cells were incubated with different treatments as appropriate. At the end of treatment, 20 μL of 5 mg/mL MTT were added to the medium that was then incubated for 4 h at 37 °C. The supernatant was removed and 150 μL of DMSO was used to dissolve the precipitate. The absorbance of the mixtures was determined at 570 nm in a plate reader (VariosKan, Thermo Fisher Scientific, Waltham, MA, USA).

### 2.4. Tube Formation Assay

The in vitro tube formation assay was carried out in Matrigel Basement Membrane Matrix system growth factor reduced (Becton and Dickinson, Milano, Italy). The experimental protocol was performed according to the manufacturer’s instructions in 96-well plates [26]. In brief, a gel solution was thawed at 4 °C overnight, then appropriate numbers of wells were coated with 50 μL of Matrigel and allowed to solidify at 37 °C for 2 h. HREC were seeded at a density of 15 × 10^3^ cells per well in 100 μL of assay medium, containing NG, HG with or without metoprolol (10 μM), epinephrine (1 μM), or with the combination of both compounds. Each condition was run in triplicate. Matrigel provided a three-dimensional support that promoted the formation of a tube-like structure network that was suggestive of in vivo capillaries. After 5 h of incubation, the tube-like structures were photographed by using an inverted microscope. The number and length of the master segments and branches were quantified with Image J software (Version 1.52a, NIH, Bethesda, MD, USA).

### 2.5. ROS Measurements

Reactive oxygen species (ROS) were measured by means of the DCFDA—Cellular Reactive Oxygen Species Detection Assay Kit (ab113851, Abcam Cambridge, UK), according to the manufacturer’s protocol [27]. After the treatments, HREC were incubated with 25 μM 2′,7′-dichlorodihydrofluorescein diacetate (DCFDA) in a buffer solution at 37 °C for 30 min. Then, DCFDA was replaced with 100 µL of medium and the ROS concentration was measured by VarioskanTM (λex = 495 nm, λem = 529 nm).

### 2.6. Western Blot Analysis

A Western blot was used to analyze the proteins that were extracted from whole cell lysates as described previously [28]. The cell lysates were produced using RIPA buffer (that was supplemented with protease and phosphatase inhibitor cocktails) (Sigma-Aldrich, St. Louis, MO, USA). Protein amounts of 30 µg were loaded on 4–20% precast polyacrylamide gel (Mini-PROTEAN^®^ TGX™ Precast Protein Gels, (Bio-Rad Laboratories, Milan, Italy) and separated by electrophoresis. The proteins were then transferred to nitrocellulose membranes. Subsequently, the membranes were blocked for 0.5 h with Odyssey Blocking Buffer (LI-COR Biosciences, Lincoln, NE, USA), and were incubated at 4 °C overnight with the antibodies against phospho-ERK 1/2 (1:500), total ERK 1/2 (1:1000), phospho-cPLA_2_ (1:500), cPLA_2_ (1:1000), Keap1 (1:1500), HO-1 (1:1500), TNF-, and Nrf2 (1:1000). GAPDH (1:1000) served as the loading control. The immunoblot was detected through an Odyssey Imaging System (LI-COR Biosciences, Lincoln, NE, USA). Densitometry analyses of the blots were performed using Image J software (National Institutes of Health, Bethesda, MD, USA).

### 2.7. High-Content Screening (HCS) and Image Analysis

High-content screening analysis was performed to evaluate the cellular distribution of ERK1/2 and cPLA_2_ cellular localization as well as Nrf2 transcription factor nuclear translocation as described previously [29]. HREC were seeded in 96-well plates at the density of 1.5 × 10^3^ and treated with NG or HG with or without metoprolol (Met, 10 µM), or epinephrine (Epi, 1 µM) for 48 h. After the treatments, the cells were washed three times with PBS 1X and were fixed with 4% paraformaldehyde for 30 min at 4 °C. Then, permeabilization and blocking were carried out by incubating the samples for 30 min in a 5% solution of normal goat serum and 0.2% Triton X in PBS 1X. Protein staining was performed by incubating the cells overnight at 4 °C with primary antibody against phospho-ERK 1/2, total ERK, phospho-cPLA_2_, total cPLA_2_, and Nrf2 at a 1:200 dilution in a PBS/0.1% triton solution. The excess of primary antibody was removed by washing the cells three times with a 0.1% Tween20 in PBS 1X solution. We then incubated the samples with both the Alexa Fluor 488 that was conjugated secondary antibody and the Cy3 conjugated secondary antibody in PBS 1X (1:1000 and 1:200 dilution, respectively) for 1 h at room temperature. Subsequently, after the cells were washed three times with PBS 1X, we stained the nuclei with Hoechst solution for 15 min at room temperature as described in the datasheet provider (Thermo Fisher Scientific, Monza, Italy). Images were acquired using the PerkinElmer Operetta High-Content Imaging System (HH12000000). The plates were analyzed in confocal conditions using the 20× long WD objective. The images were acquired using UV light, em: 460 nm, channel for Hoechst-stained nuclei, shown in blue, Alexa Fluor 488 channel (ex: 496 nm, em: 519 nm) for phospho-cPLA_2_ and total ERK1/2 staining shown in green and Cy3 (ex: 557 nm, em: 576 nm) for phospho-ERK1/2, total cPLA_2_ and Nrf2 staining shown in red. Analysis was carried out by using at least 200 cells that were captured per well. The analysis of all images and the quantification of fluorescence intensity in each cell was performed by Harmony high-content imaging and analysis software (PerkinElmer, Hopkinton, MA, USA). First, the software carried out cell segmentation in the UV channel by identifying the blue stained nuclei by screening with Area > 30 µm. Then, the cytoplasm was identified in the segmented cells based on the green staining. The mean intensity per well of Alexa Fluor 488 in the cytoplasm and nuclei was considered as phospho-cPLA_2_ and the total ERK1/2 and mean intensity per well of Cy3 in cytoplasm and nuclei was considered as phospho-ERK1/2, total cPLA_2_, and Nrf2 protein levels using the calculate intensity properties function. Nuclear translocation of Nrf2 was evaluated after nuclei identification where we considered the fluorescence intensity of Cy3 (as indicative of Nrf2 protein levels) in the nuclei population of the control HREC using the select population function. We then identified the threshold level of green fluorescence intensity up to the 95° percentile of the nuclei population in the untreated HREC. Nuclear translocation of the different experimental conditions was calculated by considering all the nuclei with a greater intensity for Cy3 staining than the threshold value as positive. Finally, the percentage of positive nuclei for Nrf2 was calculated using the calculate properties function using the formula: A/B × 100, where A was the number of positive nuclei and B was the number of analyzed objects (cells). The final output values of the nuclear translocation evaluation were reported as mean per well.

### 2.8. Extraction of Total RNA and Real-Time Reverse Transcriptase-Polymerase Chain Reaction (RT-PCR)

TRIzol reagent (Invitrogen Life Technologies, Carlsbad, CA, USA) was used to extract the total RNA from HREC according to the manufacturer’s instructions and then re-dissolved in 30 μL RNase-free water. The RNA concentration and purity were estimated by optical density that was measured at 260 and 280 nm, respectively. First-strand cDNA was synthesized by reverse transcription (RT) of 1 µg RNA in a 20 μL reaction volume with 200 U of SuperScript III, 50 ng random hexamers, 1.25 mM dNTP, 10 mM dithiothreitol, 50 mM Tris-HCl, pH 8.3, 75 mM KCl, and 3 mM MgCl_2_ (ThermoFisher Scientific, Carlsbad, CA, USA). The reaction, carried out at 50 °C for 50 min, was stopped at 85 °C for 5 min. Aliquots of 5 ng cDNA were amplified in parallel reactions using Quant Studio 3 applied biosystems, Thermo Fisher Scientific (Waltham, MA, USA). Each PCR reaction (10 μL final volume) contained 0.8 μM of forward and reverse specific primers, 1X iTaq™ Universal SYBR^®^ Green Supermix (Bio-Rad Laboratories, Milan, Italy) and 1 µL of cDNA. A total of 40 amplification cycles were carried out for each sample. The results were analyzed with the 2^−ΔΔCt^ method and were normalized by the product of 18S ribosomal RNA (rRNA) gene expression. The primers were purchased from Eurofin Genomics (Milan, Italy). The forward (Fw) and reverse (Rv) primer sequences, product size, and gene bank accession number are listed in Table 1. The specificity of the PCR reaction was assessed by the denaturation temperature of the amplification products.

### 2.9. Statistical Analysis

A total of three experiments were carried out and each experiment included four parallel samples for group (*n* = 4). The data are reported as the mean ± the standard deviation (SD). The different groups/conditions were compared by the non-parametric Mann-Whitney U-test; a *p* value < 0.05 was considered to denote a statistically significant difference between the experimental and control groups. The statistical analysis and graph design were carried out by means of GraphPad Prism 7.00 software (GraphPad Inc., San Diego, CA, USA).

## 3. Results

### 3.1. β1-Adrenergic Receptor Blockade Prevents HG-Induced Effects upon Metabolic Activity and Cell Proliferation in HREC

**Figure 1:**
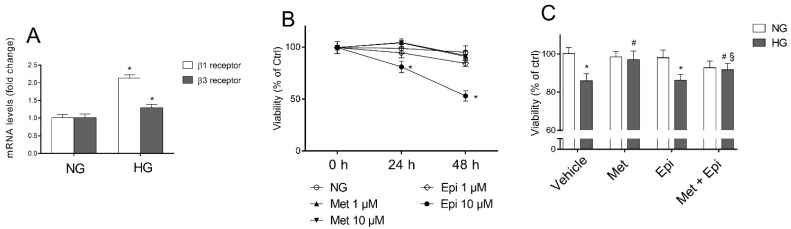
The effects of metoprolol and epinephrine on HREC that were treated with HG. (**A**) β1- and β3-adrenergic receptor mRNA levels, evaluated by qRT-PCR after 48 h of treatment with normal glucose levels (NG, 5 mM) or with high glucose levels (HG, 25 mM). (**B**) Cell viability that was evaluated by MTT assay in cells that were treated for 24 and 48 h with NG in the presence of 1 and 10 µM of metoprolol (Met), 1 and 10 µM of epinephrine (Epi). (**C**) The cell viability that was evaluated by MTT assay in cells that were treated for 48 h with NG or HG (vehicle), in cells that were treated for 48 h with NG or HG in the presence of 10 µM of metoprolol (Met), or 1 µM of epinephrine (Epi), or in the presence of both compounds (Met + Epi). The values are expressed as the mean ± SD of three independent experiments with four parallel samples for group in each experiment. * *p* < 0.05 vs. NG; ^#^ *p* <0.05 vs. HG; ^§^ *p* < 0.05 vs. HG-Epi. The non-parametric Mann-Whitney test was used for pairwise comparisons.

To elucidate the involvement of β-AR-mediated signaling in cell damage that was induced by hyperglycemia, we first evaluated the mRNA levels of β1-, β2-, and β3-AR in HREC that was treated with 25 mM of HG to mimic in vitro the insult to the retinal endothelium occurring in late stage of DR. As indicated in Figure 1 panel A, after HG treatment for 48 h, β1-AR mRNA levels were nearly double and β3-AR mRNA was 1.3-fold higher compared with the respective levels in the untreated cells. The β2-AR mRNA level was undetectable in HREC (data not shown). 

The effects of metoprolol (β1-AR antagonist) and/or epinephrine (β-AR agonist) were tested in HREC that was stimulated with HG (panel C). Preliminarily, the experiments were carried out to assess the subtoxic concentrations for metoprolol and epinephrine in HREC that was incubated in the presence of normal glucose concentrations (panel B). The evaluation of the mitochondrial function (cell viability) of HREC was assessed by MTT assay after treatment with 1 and 10 μM of these compounds for 24 and 48 h. Neither metoprolol nor epinephrine treatment at 1 μM changed the HREC viability after incubation for 24 or 48 h in comparison to the untreated cells, whereas epinephrine at 10 μM induced a reduction of cell viability by about 50%. In panel C, the cell viability data that was observed in HREC that were treated for 48 h with 10 μM of metoprolol, 1 μM of epinephrine, or their association, in presence of NG or HG, are shown. As expected, HG induced a reduction of cell viability by about 18% compared with NG [26]. The presence of metoprolol significantly increased cell viability up to control levels (HG-treated HREC). Epinephrine reduced the HG-treated HREC viability by 11% and metoprolol restored the epinephrine-affected viability in high glucose conditions.

### 3.2. Metoprolol Counteracts the Increase in the Tube-like Structures of HREC Stimulated with HG

We further examined the tube formation capability of HREC that was stimulated by HG in the presence of 10 μM of metoprolol, 1 μM of epinephrine, or both compounds. A tube formation assay was carried out in Matrigel that provide the endothelial cells with a three-dimensional scaffold for the production of a network of interconnecting tube-like structures, representing a suggestive in vitro model of HG-induced angiogenesis [30,31].

**Figure 2:**
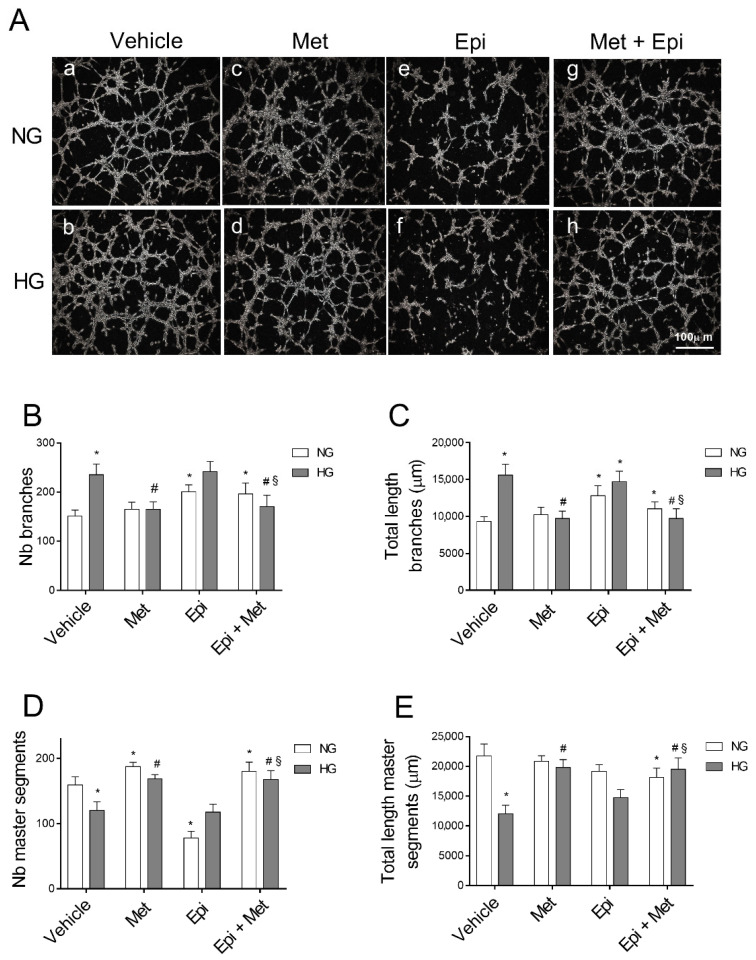
The role of metoprolol and epinephrine in the tube-like structures that were assessed by the tube formation assay in HREC that was stimulated with HG. HREC were seeded into 96-well plates that were coated with Matrigel at a density of 1.5 × 10^4^/well. In the presence of (a) normal glucose levels (NG, 5 mM) or (b) high glucose levels (HG, 25 mM), supplemented with metoprolol (Met, 10 µM) in absence (c) or in presence (d) of high glucose, epinephrine (Epi, 1 µM) in absence (e) or in presence (f) of high glucose, or both compounds in absence (g) or in presence (h) of high glucose. The representative microscopic field (100× magnification) showing the tube-like structures that were acquired after 4 h of cell seeding (**A**). The images were analyzed with Image J software to quantify the total number (**B**) and length (**C**) of the branches as well as total number (**D**) and length (**E**) of the master segments of the tube-like structures. The values are expressed as mean ± SD of three independent experiments with four parallel samples for group in each experiment. * *p* < 0.05 vs. vehicle in NG conditions; ^#^ *p* < 0.05 vs. HG; ^§^ *p* < 0.05 vs. HG-epinephrine. The non-parametric Mann-Whitney test was used for pairwise comparisons.

As shown in Figure 2A, HG treatment deeply modulated the tube formation capability of HREC (b). In fact, quantitative analyses of the tube-like structures (Figure 2B,C) showed that HG significantly enhanced the number and total length of branches (by about 57% and 67%, respectively) and reduced the number of master segments and their total length by about 24% and 45%, respectively (Figure 2D,E) in comparison to NG-treated cells. Mannitol (25 mM) showed no effect on the formation of the tube-like structures (data not shown). Treatment with metoprolol 10 µM in NG did not affect the formation of the tube-like structures in comparison to the vehicle (Figure 2A(c)), while it significantly prevented the tube-like structure formation (Figure 2A(d)) and the alterations in the number and total length of the branches (reduced by 30% and 37%, respectively) and in the number and total length of the master segments (increased by 40% and 64%, respectively) in the presence of HG (Figure 2B–E), restoring the values to those that were observed in NG (control condition).

In the presence of HG, 1 µM of epinephrine had no effect in comparison to the corresponding conditions in its absence (Figure 2A(e,f)), as confirmed by the number and total length of the branches when compared with the control cells (Figure 2B,C), whereas, in NG conditions, the branch number increased by 33% in the presence of epinephrine in comparison to the respective controls. Epinephrine significantly impaired angiogenesis in HREC, reducing the number of master segments in NG by 51% in comparison to the control cells (Figure 2D). Moreover, a slight increase in the total length of the master segments was induced in HREC by epinephrine treatment in HG conditions in comparison to the vehicle (panel E). Moreover, in the presence of NG, the co-treatment of the cells with metoprolol and epinephrine did not show any significant impairment in the tube-like structures compared with HG-epinephrine (Figure 2A(g,h)). However, in the cells that were treated with metoprolol and epinephrine, the presence of metoprolol completely suppressed the effect of epinephrine in HG conditions. The co-treatment with epinephrine and metoprolol in HG conditions reduced both the number and the total length of the branches by 30% and 34%, respectively, in comparison to the epinephrine treated cells (Figure 2B,C). The co-treatment, both in NG and HG conditions, increased the number of master segments and their total length by 2.3 and 1.4-fold, respectively, in comparison to HREC in the presence of epinephrine. The co-treatment in HG was also able to increase the total length of the master segments by 32% with respect to the HREC that was treated with epinephrine, restoring the values to those that were observed in NG. These data suggested that β1-AR blockade by metoprolol could modulate vascular remodeling that is driven by HG in HREC.

### 3.3. Metoprolol Down-Regulates the ERK1/2/cPLA_2_/COX2 Axis in HREC Treated with HG

**Figure 3:**
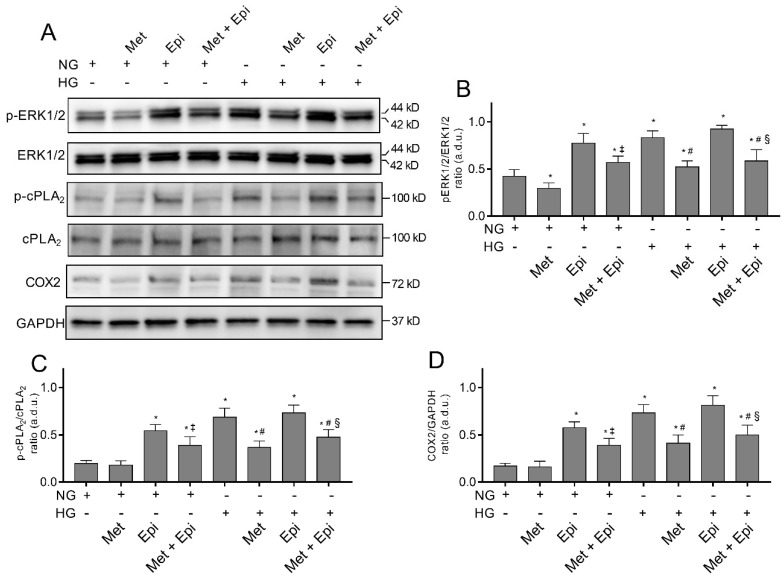
The effects of metoprolol and epinephrine on the activation of the pro-inflammatory ERK1/2/cPLA_2_/COX2 pathway as assessed by a Western blot in HREC that was treated with HG. The proteins were extracted from whole lysate of cells that were treated in the presence of normal glucose levels (NG, 5 mM) or high glucose levels (HG, 25 mM) alone, or supplemented with metoprolol (Met, 10 µM), epinephrine (Epi, 1 µM), or both compounds. (**A**) Immunoblot analysis that was performed using specific antibodies against phospho- and total ERK1/2, phospho- and total cPLA_2_, and COX2. The blots were probed with anti-GAPDH antibody to verify equal loading of 30 µg protein per lane. Image J software was used to carry out densitometric analysis of immunoblots indicating protein quantification of each band (in arbitrary densitometry units, a.d.u.). The graphs report the phospho/total band intensity ratio that was normalized with respect to the corresponding reference for ERK1/2 (**B**), cPLA_2_ (**C**), and COX2 (**D**). Protein induction was documented as COX2/GAPDH band intensity ratio. The data are representative of three independent experiments with four parallel samples for group in each experiment and are expressed as the mean ± SD. * *p* < 0.05 vs. NG; ^‡^ *p* < 0.05 vs. NG-epinephrine; ^#^ *p* < 0.05 vs. HG; ^§^ *p* < 0.05 vs. HG-epinephrine. The non-parametric Mann-Whitney test has been used for pairwise comparisons.

Hyperglycemia has been shown to trigger cell damage in the retinal endothelium by activating the ERK/cPLA_2_/COX-2/PGE2 axis, both in vivo and in vitro [32,33]. Moreover, the activation of this pro-inflammatory pathway is an event that occurs in the endothelial cells and pericytes after stimulation with HG; this event is related to increased VEGF-A expression [13,24]. We tested the hypothesis that β-adrenergic signaling could modulate the pathway, thus providing molecular insight regarding the protective effects of the β1-AR blockade in HG-stimulated HREC. An immunoblot analysis was used to assess the amount of ERK1/2, cPLA_2_, and COX2 in lysates from HREC that was treated with NG or HG that was supplemented with 10 μM of metoprolol, with 1 μM of epinephrine, or with both compounds. As shown in Figure 3, we observed that the treatment with HG 25 mM increased the phosphorylated ERK1/2 and cPLA_2_ levels by about 2- and 3-fold, respectively (*p* < 0.05), as well as enhancing COX2 protein levels (by about 4-fold, *p* < 0.05) in comparison to NG, indicating the HG-induced activation of this pro-inflammatory pathway.

The presence of metoprolol in NG conditions decreased the basal levels of phospho-ERK1/2 (NG-treated cells) by about 30% and reduced the HG-induced up-regulation of phospho-ERK1/2, phospho-cPLA_2_, and COX2, almost completely restoring the control levels. In NG conditions, epinephrine increased the phospho-ERK1/2 levels by about 65% as well as phospho-cPLA_2_ and COX2 (by about 3-fold, *p* < 0.05) compared with the NG cells. However, in the presence of HG, epinephrine did not significantly change either the phospho-ERK1/2 or phospho-cPLA_2_ and COX2 protein levels. The co-treated HREC with metoprolol and epinephrine in the presence of NG showed a partial reduction in phospho-ERK and COX2 protein levels (of about 2-fold, *p* < 0.05) compared with the cells that were treated with epinephrine. The co-treatment with metoprolol and epinephrine in the presence of HG reduced phospho-ERK, phospho-cPLA_2_, and COX2 protein levels in comparison to the HG-stimulated cells that were treated with epinephrine (Figure 3A–D). All the treatments did not change the protein levels of total ERK1/2 and cPLA_2_. The combination of agonist and antagonist in HG conditions highlighted the effect of metoprolol in reducing ERK1/2, cPLA_2_, and COX2 activation.

Overall, these results indicated that β-adrenergic signaling modulates the activation of the pro-inflammatory ERK/cPLA_2_/COX-2 pathway; β1-AR blockade by metoprolol exerts its main activity in the prevention of the inflammatory pathway activation in HG-stimulated HREC.

### 3.4. Metoprolol Reduced cPLA_2_ Nuclear Translocation in HREC Challenged with HG

**Figure 4:**
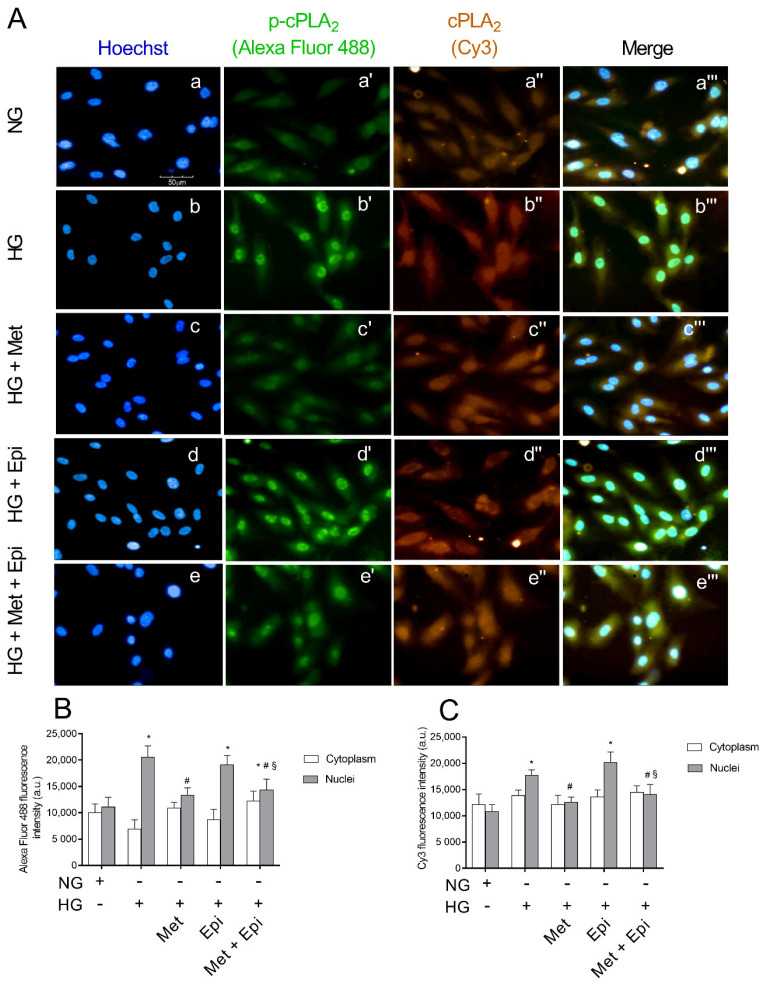
The effects of metoprolol and epinephrine on the nuclear translocation of cPLA_2_ as assessed by immunocytochemistry in HREC that was treated with HG. (**A**) Representative images of immunocytochemical staining for phospho-cPLA_2_ (p-cPLA_2_) and total cPLA_2_ (cPLA_2_) in HREC. In presence of normal glucose, Hoechst-stained cell nuclei (a), p-cPLA_2_ green fluorescence (a′), cPLA_2_ red fluorescence (a″) and merged pictures (a‴). In presence of high glucose, Hoechst-stained cell nuclei (b), p-cPLA_2_ green fluorescence (b′), cPLA_2_ red fluorescence (b″) and merged pictures (b‴). In presence of high glucose and metoprolol, Hoechst-stained cell nuclei (c), p-cPLA_2_ green fluorescence (c′), cPLA_2_ red fluorescence (c″) and merged pictures (c‴). In presence of high glucose and epinephrine, Hoechst-stained cell nuclei (d), p-cPLA_2_ green fluorescence (d′), cPLA_2_ red fluorescence (d″) and merged pictures (d‴). In presence of high glucose plus metoprolol and epinephrine, Hoechst-stained cell nuclei (e), p-cPLA_2_ green fluorescence (e′), cPLA_2_ red fluorescence (e″) and merged pictures (e‴). The HG conditions increased the green fluorescence particularly in the nuclear region compared with the control HREC (b′ vs. a′) indicating cPLA_2_ nuclear translocation; this effect was reduced by metoprolol (c′ vs. b′) but not by epinephrine (d′ vs. b′). Magnification: ×40; scale bars: 100 µm. Each value represents the mean ± SD that was obtained from three independent experiments with four parallel samples for group in each experiment (**B**,**C**). * *p* < 0.05 vs. NG nuclei; # *p* < 0.05 vs. HG nuclei; ^§^ *p* < 0.05 vs. Epi nuclei. The non-parametric Mann-Whitney test was used for pairwise comparisons.

Immunofluorescence microscopy analyses confirmed the enhanced activation of cPLA_2_ in HREC after treatment with HG. The expression of total cPLA_2_ and its phosphorylated and active form phospho-cPLA_2_ is shown in Figure 4. The intensity of fluorescence was obtained separately with FITC (p-cPLA_2_) and CY3 (cPLA_2_) filters. Merges of the images are also shown. It is evident that cPLA_2_ was constitutively expressed in HREC (A, panel a″) and phospho-cPLA_2_ was uniformly distributed within the cells (A, panel a′). Images that related to the HG treatment displayed very strong immunofluorescence signals that related to the activated phospho-cPLA_2_, which is particularly evident at the nuclear level, indicating the migration of the enzyme from the cytosol to the nuclear membranes (A, panel b′). Moreover, the total cPLA_2_ immunofluorescence was evident at the nuclear level after HG-treatment (panel b″). Migration and activation of phospho-cPLA_2_ in HG-treated cells was enhanced in the merge of the three panels (panel b‴) with respect to the corresponding merge of the untreated cells (panel a‴). Following the treatment with HG, the presence of metoprolol markedly decreased the fluorescence intensity relative to phospho-cPLA_2_ (panel c″), whereas in presence of epinephrin, the fluorescence intensities relative to cPLA_2_ and phospho-cPLA_2_ (panels d′ and d″) were markedly higher than in the unstimulated control cells (panel a and b). A merge of the two images shows an enhanced expression of both enzymatic forms (panel d″). The emission intensities of FITC and Cy3 were also evaluated and the pixel values inside the cells on a scale of 0–25,000 pixels (fluorescence arbitrary units) were reported in the graph for quantitative analysis (B and C). After treatment of HREC with HG, the fluorescence that related to phospho-cPLA_2_ inside the nuclei increased by 1.81-fold compared with the untreated cells. Metoprolol induced a reduction of the fluorescence to values very similar to the control cells with a decrement of 35% respect to HG-HREC. The incubation with HG in the presence of epinephrine induced an increase of nuclear fluorescence that was related to phospho-cPLA_2_ of almost 1.73-fold compared with the untreated cells. The presence of metoprolol in HG-epinephrine-treated cells significantly reduced the phospho-cPLA_2_ levels in the nuclei (reduction by 27%). In panel C, the distribution of the total cPLA_2_ showed the same trend as the phosphorylated form of the enzyme.

### 3.5. Metoprolol Reduced ERK 1/2 Phosphorylation in HREC Challenged with HG

**Figure 5:**
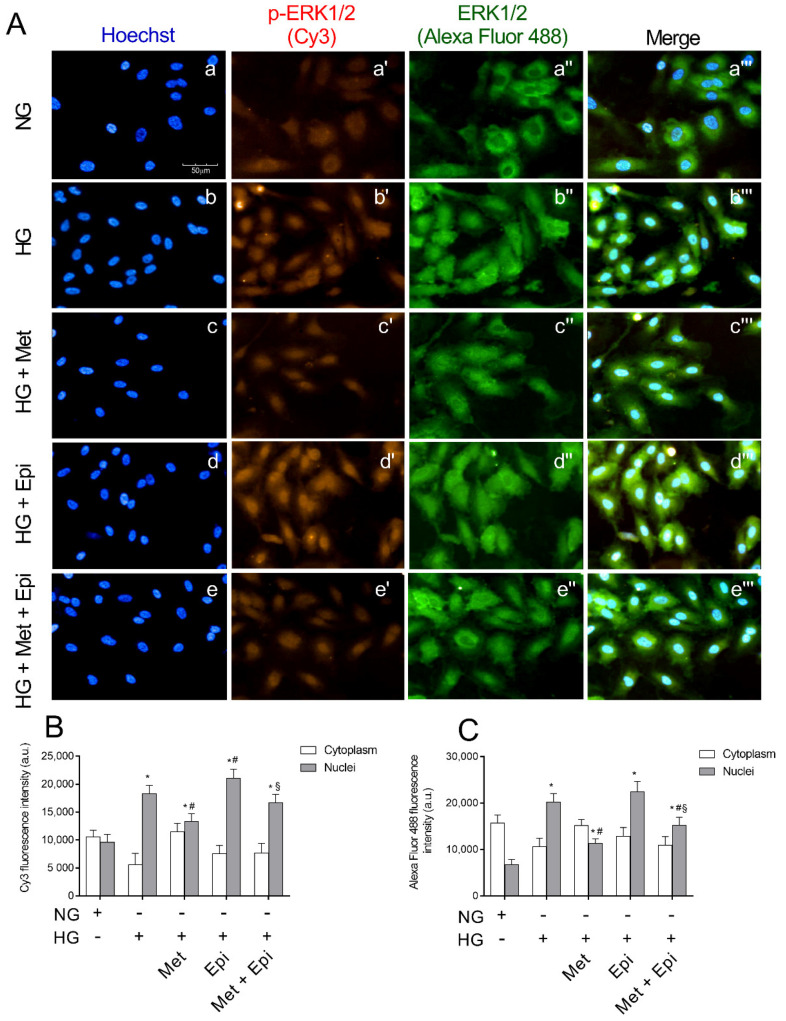
The effects of metoprolol and epinephrine on ERK1/2 nuclear translocation as assessed by immunocytochemistry in HREC that was treated with HG. (**A**) Representative images of immunocytochemical staining for phospho-ERK1/2 (p-ERK1/2) and total ERK1/2 (ERK1/2,) in HREC. In presence of normal glucose, Hoechst-stained cell nuclei (a), p-ERK 1/2 red fluorescence (a′), ERK 1/2 green fluorescence (a″) and merged pictures (a‴). In presence of high glucose, Hoechst-stained cell nuclei (b), p-ERK 1/2 red fluorescence (b″), ERK 1/2 green fluorescence (b″) and merged pictures (b‴). In presence of high glucose and metoprolol, Hoechst-stained cell nuclei (c), p-ERK 1/2 red fluorescence (c′), ERK 1/2 green fluorescence (c″) and merged pictures (c‴). In presence of high glucose and epinephrine, Hoechst-stained cell nuclei (d), p-ERK 1/2 red fluorescence (d′), ERK 1/2 green fluorescence (d″) and merged pictures (d‴). In presence of high glucose plus metoprolol and epinephrine, Hoechst-stained cell nuclei (e), p-ERK 1/2 red fluorescence (e′), ERK 1/2 green fluorescence (e″) and merged pictures (e‴). HG conditions increased the red fluorescence, particularly in the nuclear region compared with the control HREC (b′ vs. a′) indicating ERK1/2 nuclear translocation; this effect was reduced by metoprolol (c′ vs. b′) but not by epinephrine (d′ vs. b′). Magnification: ×40; scale bars: 100 µm. Each value represents the mean ± SD obtained from three independent experiments with four parallel samples for group in each experiment (**B**,**C**). * *p* < 0.05 vs. NG nuclei; ^#^ *p* < 0.05 vs. HG nuclei; ^§^ *p* < 0.05 vs. Epi nuclei. The non-parametric Mann-Whitney test was used for pairwise comparisons.

In Figure 5, the images of immunocytochemical staining for ERK1/2 and its phosphorylated form are shown. The intensity of fluorescence was obtained separately with FITC (ERK1/2) and CY3 (pospho-ERK1/2) filters. After incubation for 48 h with HG, an increase of fluorescence intensity that was relative to phospho-ERK1/2 was detected (panel b′) compared with the untreated cells (panel a′). The incubation with metoprolol, during the treatment with HG reduced the red fluorescence (panel c′), indicating that the antagonist was able to block ERK1/2 phosphorylation. In the presence of epinephrine, ERK1/2 activation was highlighted by the increase of the fluorescence intensity (panel d′). The pixel values inside the cells, as reported in panel B, showed an increase of phospho-ERK1/2 in the nuclei of HREC that were treated with HG by about 1.9-fold in comparison to the untreated cells. The presence of metoprolol in the incubation medium with HG induced a reduction of the fluorescence by almost 30% in comparison to HG-HREC; the presence of epinephrine in HG-HREC induced a slight increase (1.15-fold) in the distribution in nuclei of the phosphorylated protein in comparison to the HG-treated cells. The presence of metoprolol in the HG-Epi-treated cells significantly reduced phospho-ERK1/2 levels in nuclei by 21% in comparison to the HG-Epi-treated cells.

Moreover, in HG-HREC, the total ERK in the nuclei increased by 3.0-fold with respect to the NG-treated cells and metoprolol significantly reduced the fluorescence that was related to the nuclei by 44% (panel C). Again, in HG-epinephrine-treated cells, metoprolol reduced ERK1/2 levels in nuclei by 32% in comparison to the HG-epinephrine-treated cells. As such, metoprolol reduced total ERK shift from the cytoplasm to the nuclei in both HG- and HG-epinephrine-treated cells.

### 3.6. Metoprolol Down-Regulates the Release of TNF-α, VEGF, and IL-1b in HREC Treated with HG

**Figure 6:**
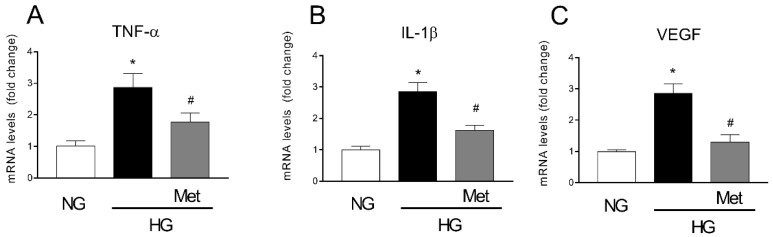
The effects of metoprolol on the pro-inflammatory cytokines and VEGF-A factors in HREC that was stimulated with HG. The cells were cultured in NG (5 mM) or in HG (25 mM) alone or supplemented with metoprolol (Met, 10 µM), epinephrine (Epi, 1 µM), or both compounds. The data were gathered after 48 h from treatment addition. Quantitative analysis of TNF-α (**A**), IL-1β (**B**), and VEGF (**C**) mRNA levels, as evaluated by qRT-PCR. The mRNA levels of each group were normalized to the housekeeping reference gene ribosomal 18S RNA. In the histograms, the values are expressed as fold change of those that were detected in HREC that was cultured in NG condition. Each value represents the mean ± SD that was obtained from three independent experiments with four parallel samples for group in each experiment. * *p* < 0.05 vs. NG; ^#^ *p* < 0.05 vs. HG. The non-parametric Mann-Whitney test was used for pairwise comparisons.

Since the data that were obtained showed that the β1-AR blockade could prevent vascular remodeling as well as the activation of the pro-inflammatory ERK1/2/cPLA_2_/COX2 pathway induced by HG in HREC, we aimed to investigate whether the metoprolol effects could cause a modulation of TNF-α, IL-1β, and VEGF mRNA levels in HREC that was treated with NG (5 mM) or HG (25 mM) with or without 10 μM of metoprolol. As shown in Figure 6, the treatments with HG significantly increased the mRNA levels of TNF-α, IL-1β, and VEGF by about 3-fold (*p* < 0.05). The presence of metoprolol in HG conditions prevented the HG-induced increase in the mRNA levels of TNF-α, IL-1β, and VEGF by about 39%, 43%, and 54%, respectively (*p* < 0.05). These data suggested that the anti-inflammatory and anti-angiogenic activity of metoprolol is coupled with the reduction of TNF-α, IL-1β, and VEGF genes.

### 3.7. Metoprolol Counteracts Glucose-Induced ROS Accumulation by Activating the Keap1/Nrf2/HO-1 Pathway in HREC

**Figure 7:**
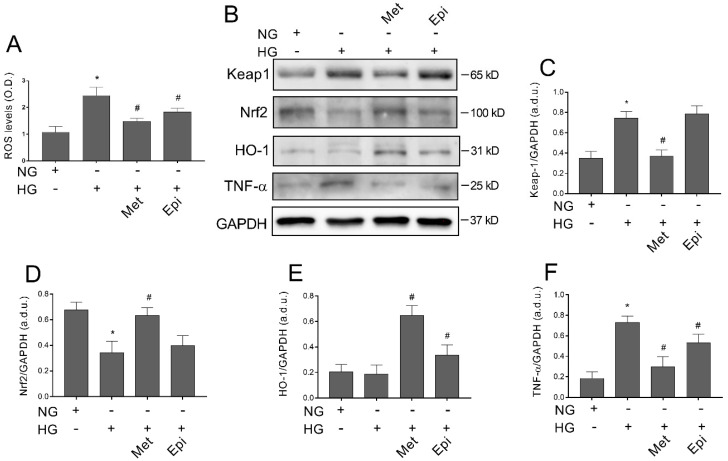
Anti ROS activity of metoprolol and Keap1/Nrf2/HO-1 axis activation in HREC that was treated with HG. The cells were treated in the presence of normal glucose levels (NG, 5 mM), or high glucose levels (HG, 25 mM) alone or supplemented with metoprolol (Met, 10 µM) and epinephrine (Epi, 1 µM) for 48 h. (**A**) ROS levels as evaluated by H2DCFDA assays. (**B**) Immunoblot analysis was performed using specific antibodies against Keap1, Nrf2, HO-1, and TNF proteins. The blots were probed with anti-GAPDH (reference) antibody to verify the equal loading of 30 µg protein per lane. Image J software was used to carry out densitometric analysis of the immunoblots, indicating protein quantification of each band (in arbitrary densitometry units, a.d.u.). The graphs show the target/GAPDH band intensity ratio that was normalized with respect to the corresponding reference for Keap1 (**C**), Nrf2 (**D**), HO-1 (**E**), and TNF-α (**F**). The data are representative of three independent experiments with four parallel samples for group in each experiment and are expressed as mean ± SD. * *p* < 0.05 vs. NG; ^#^ *p* < 0.05 vs. HG. The non-parametric Mann-Whitney test was used for pairwise comparisons.

ROS accumulation represents a key event in hyperglycemia-induced cell damage. Moreover, it has been described that Keap1/Nrf2 pathway inhibition that is elicited by high levels of glucose strongly contributes to ROS increase in the retinal endothelium [22,23]. We aimed to elucidate whether metoprolol could have an impact on high glucose level-induced ROS accumulation and its underlying mechanisms. For this purpose, we tested the effect of the metoprolol in HREC that was treated with HG for 48 h on ROS production, evaluated by H2DCFDA. The data that are shown in Figure 7A indicate that HG increased ROS production by about 2.4-fold (*p* < 0.05) compared with the NG-treated cells. The presence of metoprolol prevented ROS production, with a reduction by 38% (*p* < 0.05). A reduction of 24% on ROS production was also induced by epinephrine with respect to the HG-induced ROS, confirming its potential free radical scavenging activity [34]. Therefore, in our model, we tested the hypothesis that metoprolol could activate the Keap1/Nrf2/HO-1 pathway to provide molecular insight regarding its anti-ROS activity. As can be seen in Figure 7, a Western blot analysis showed that HG increased Keap1 (panel C) and TNF-α (panel F) by about 2.2- and 4-fold, respectively, (*p* < 0.05), and reduced Nrf2 protein levels (panel D) by about 50% (*p* < 0.05) compared with the NG cells. The presence of 10 μM of metoprolol decreased Keap1 protein content to the control levels, as well as TNF-α and increased the Nrf2 production to values that were very similar to those of the untreated cells. The presence of epinephrine in the HG-treated cells induced no effect on Keap1, Nrf2, and TNF-α expression in comparison to the HG-cells in absence of the agonist. The treatment with HG did not change, instead, the expression of HO-1 and the presence of metoprolol or epinephrine induced an increase of the expression by 3.5- and 1.7-fold, respectively, in comparison to the HG-treated cells.

These data indicate that metoprolol is able to counteract ROS accumulation in HREC that is stimulated by HG by activating the Keap1/Nrf2/HO-1 pathway and by inhibiting ROS production.

### 3.8. Metoprolol Induced Nuclear Nrf2 Nuclear Compartmentalization in HG-Treated HREC

**Figure 8:**
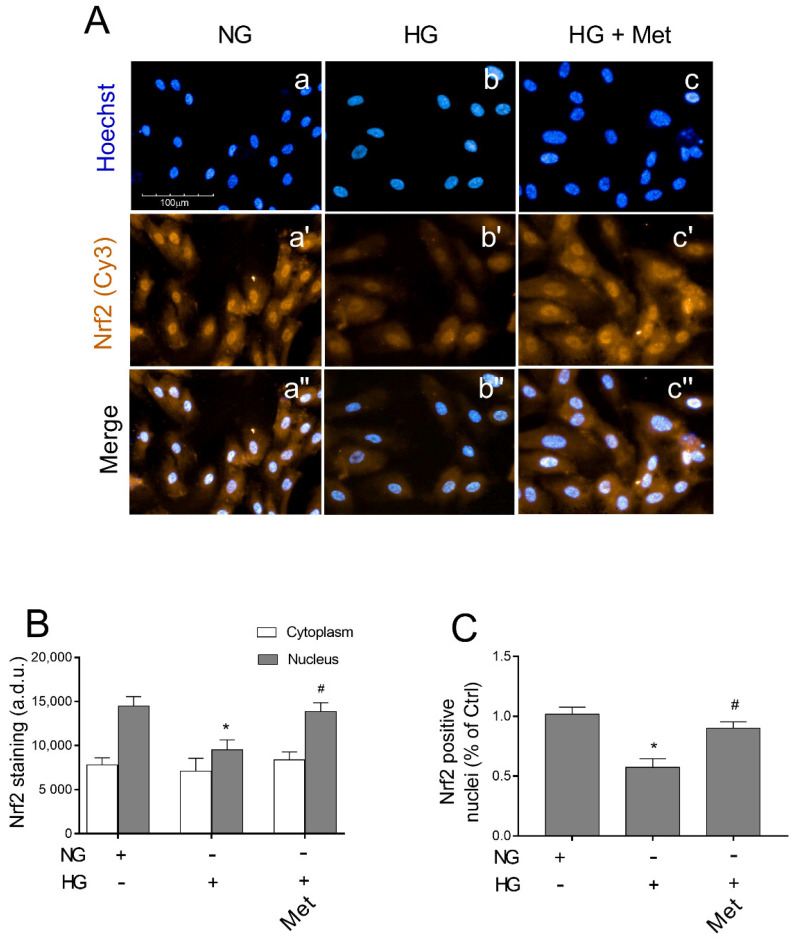
The effects of metoprolol on Nrf2 nuclear translocation in HREC that was stimulated with high glucose levels. (**A**) The representative of immunocytochemically stained images for Nrf2 in HREC that was cultured in normal glucose levels (NG, 5 mM) or in HG, (25 mM) alone or supplemented with metoprolol (Met, 10 µM) for 48 h (in red: a′–c′ respectively). Moreover, the relative Hoechst-stained nuclei (a–c, respectively) and merged channels (a″–c″, respectively) are shown. All images were acquired with the Operetta High-Content Imaging System using a 20× magnification; scale bar = 100 µm. (**B**) High-content screening analysis was used to quantify the green fluorescence (Cy3) relative to the Nrf2 staining in the cytoplasm and nuclei. (**C**) Quantification of Nrf2 positive nuclei. The data are expressed as the mean ± SD from at least six fields/well randomly selected and each reporting more than 15 cells/field. All of the data represent the mean ± SD that were obtained from at least three independent experiments with four parallel samples for group in each experiment. * *p* < 0.05 vs. NG; ^#^ *p* < 0.05 vs. HG. The non-parametric Mann-Whitney test was used for pairwise comparisons.

As metoprolol exerted protective effects on HREC that was treated with HG, inducing the activation of the Keap1/Nrf2/HO-1 pathway, we further evaluated its capability to stimulate Nrf2 nuclear localization. Immunofluorescence assays that were performed by high-content screening analysis showed a basal level of immunoreactivity for Nrf2 protein (green fluorescence) in NG-cultured cells both in the cytoplasm and nuclei (Figure 8A, panel a′). Treatments for 48 h with HG strongly reduced the green fluorescence that was observed in the nuclei (panel b′ vs. a′) where about a 34% reduction in immunoreactivity was observed compared with the NG-HREC (Figure 8B,C). The presence of metoprolol in HG conditions restored Nrf2 immunoreactivity in the nuclei of the HG-treated HREC to the control levels (*p* < 0.05) as indicated by the increased nuclear fluorescence intensity (picture c′ vs. b′) by 46% in comparison to the HG-treated cells without the agonist (Figure 8B). Indeed, compared with the controls, HG-treated HREC showed reduced percentages of about 57% (*p* < 0.05) in positive nuclei; the effect was strongly prevented by metoprolol in HG conditions, with an increase by 58% in comparison to the HG-treated cells in the absence of the agonist (Figure 8C). Overall, these results suggest that ROS accumulation in HREC that is stimulated by HG is mediated by the Keap1/Nrf2/HO-1 pathway down-regulation.

## 4. Discussion

In DR, pericyte loss leads to micro-aneurysms and, as the disease progresses, to angiogenesis that is related to HREC proliferation [35]. In fact, BRB integrity is maintained by the presence of pericytes that, by enveloping the endothelial cells, control their proliferation [24]. It has been demonstrated that β-ARs participate in the response of HREC and HRPCs to HG damage [36]. A very interesting study highlighted the involvement of β1- and β2-AR in the processes that lead to the detachment of pericytes from the retinal micro-vessels, showing that β2 mRNA was much higher than β1-AR in HRPC, and that β2-AR agonists were able to induce PC survival by ERK 1/2 phosphorylation [36].

Β-AR antagonists have recently been found to provide a therapeutic effect by counteracting several eye disorders as DR in a in vivo model [37], oxygen induced retinopathy (OIR) in a mouse model [38], and age-related macular degeneration in rabbit eyes [39]. In the OIR model, the β1-/β2-AR non selective antagonist propranolol counteracted the retinal microvascular angiogenesis in response to hypoxia in mice [40]. In the same OIR model, in retinas from knockout mice (ADRB 1/2 null mice) a protective effect, by preventing neovascularization, was demonstrated compared with the wild type [41].

Moreover, in a mouse model of OIR, β-AR blockade with non-selective antagonist propanol reduced the hypoxia-upregulated VEGF and decreased the HIF-1α levels, ameliorating the retinopathy score [42]. The authors also demonstrated that the levels of β3-AR in hypoxic retinas were significantly higher than those in the control retinas, whereas β1- and β2-AR were not significantly different from the control values, focusing on the role of β2-AR rather than β1- and β2-AR in angiogenesis occurring during OIR [42].

The different response of β-ARs to agonist and antagonist molecules in the study of both pathologies, OIR and DR, that were characterized by a similar angiogenic response, could be due to the use of different experimental models which reproduce different stages of the endothelial response.

However, in this firmament that is dotted with numerous and often conflicting data, the principal role played by β1- and β2-ARs and the supporting role of β3-AR in the enhancement of an angiogenic response that is already underway has been demonstrated in a knockout mouse model [41].

In the present study we initially evaluated the response of the three receptors after treatment of HREC with HG. Moreover, we demonstrated that β1-AR but not β3-AR-mRNA levels significantly increased after HG treatment compared with the levels in the untreated cells and that β1-AR blockade prevented the HG-induced effects on the metabolic activity of HREC. Moreover, in our model, β2-AR mRNA was not detectable both under normal and HG conditions, confirming the absence of this receptor in HREC [43].

Even in the context of the DR study, conflicting results were reported on the role played by β-ARs. For instance, in vivo experiments demonstrated that the retinal disfunction in diabetic rats was not ameliorated by treatment with the β1 antagonist atenolol, both from the point of view of the response to the electroretinogram [44] and the inhibition of angiogenesis [45]. On the other hand, a recent study compared the effects of five β-blockers (propranolol, metoprolol, atenolol, bisoprolol, and nebivolol) on the response of endothelial cells to HG and demonstrated that metoprolol, rather than other antagonists, protected the endothelial cells both in vitro and in vivo, by making wound healing faster in diabetic mice [46].

In our results, the inhibitory effect of metoprolol on the angiogenic potential of HREC in HG, highlighted with the Matrigel tube formation assay, restored the viability and tube formation capability, confirming the role of β1-AR in HG-induced endothelial injury. Metoprolol was effective in counteracting the increase in the tube-like structures of HREC that was stimulated with HG by reducing the number of branches and by increasing the number of master segments. Epinephrine was used as β-AR agonist [47,48] to validate the effect of metoprolol.

The results that were obtained here confirmed the key role of cPLA_2_ in the progression of DR since it mediates the HG-induced damage of HREC [13]. Increased cPLA_2_ activation after HG depends on ERK 1/2 phosphorylation and is responsible for the increase of COX-2, which releases PGE2, mediating the inflammatory response to endothelial cell damage that is induced by HG [26]. The β-AR agonist epinephrin induced ERK1/2-PLA_2_ axis activation, whereas the β1-AR antagonist metoprolol prevented the ERK-signaling cascade. We previously demonstrated that in vitro HG conditions increased the VEGF-A levels in HREC and this event exerted an autocrine effect stimulating inflammation, that was also mediated by PLA_2_ activation [26]. These data confirmed the evidence of the role of β-AR signaling in VEGF release, which promotes angiogenic processes as demonstrated by experiments that were conducted on endothelial cells that were obtained from proliferating infantile hemangioma and on HUVEC after treatment with a non-selective β-blocker [49,50].

It has been demonstrated that VEGF induces BRB breakdown by the activation of the protein kinase-C (PKC) pathway [51] leading to an increased phosphorylation of occludin and its internalization with the consequent BRB breakdown [52].

The in vitro model that was used in this study, based on a single treatment of HREC with HG for 48 h, mimics the late phase of diabetic retinopathy, during which the release of VEGF-A increases without inducing a significant effect on endothelium proliferation (data not shown). The prolonged and fluctuating glucose exposure for several days, based on molecular mechanisms, leads to a significative increase in endothelial proliferation, and in vitro mimics the conditions that occur in vivo that characterize proliferative DR [13].

In DR, the morphological and functional damage to the microvascular endothelial cells is caused by the release of pro-inflammatory cytokines, by the activation of TNF-α signaling, and by the triggering of oxidative stress [53]. It has been demonstrated that the treatment of retinal endothelial cells with HG increased the release of IL-1β with a mechanism involving the activation of NF-kB, suggesting a role for IL-1β in the development of retinopathy in diabetes [54]. The relationship between TNF-α and DR has been demonstrated by a meta-analysis study, indicating that the TNF-α levels were significantly different in DR patients vs. the healthy controls, and that it may be used as a biomarker in DR [55]. Moreover, hyperglycemia leads to increased ROS production in retinal micro-vessels, which are consequently particularly vulnerable to oxidative stress [56]. Our results confirmed the increased production of IL-1β and TNF-α and we provide the first evidence that their release could be modulated by β1-AR blocker during HG treatment of HREC.

The high-energy demands and the constant exposure to light makes the retina particularly susceptible to ROS and oxidative stress plays a key role in the development of DR [57]. AA, introduced with our daily diet and synthesized from linoleic acid, is incorporated at the *sn*-2 position of the glycerophospholipids of plasma membranes. After inflammatory stimuli, PLA_2_ releases AA, by cutting it from membrane phospholipids and makes it available for the catalytic action of COX and LOX, in a pathway leading to eicosanoid formation, which contributes to oxidative stress [58,59].

COX enzymes are present in two iso-enzymatic forms, COX-1 and COX-2. COX-1, the constitutive isoform, releases low concentrations of PGs which maintain cell homeostasis, whereas COX-2, the inducible isoform, releases PGE2 when the cell is stimulated by cytokines and growth factors during chronic inflammation [58]. PGE2 increases oxidative stress, vascular permeability, and the production of proinflammatory cytokines in micro-vessels [60]. It has been demonstrated in vascular endothelial cells that COX activity and expression can be modulated by ROS [61] and that the production of ROS can be regulated by PG in the pig pulmonary artery [62]. Moreover, ROS formation can be increased by PGE2 via the EP1 receptor, leading to endothelial dysfunction [63]. In our model, metoprolol exerted a marked activity in counteracting ROS accumulation in HREC that was treated with HG. This is the first evidence which correlates the anti-ROS activity of metoprolol with the activation of the Keap1/Nrf2/HO-1 pathway in HREC that is treated with HG. In fact, we observed a significant increase of HO-1 protein levels as a consequence of metoprolol treatment in the presence of HG. In our model, metoprolol was able to reduce Keap1 protein levels which naturally retain sequestered Nrf2 in the cytoplasm and prevent its function.

Moreover, we showed that metoprolol promotes the nuclear translocation of Nrf2 and we tested the beneficial effect of Keap1/Nrf2/HO-1 activation by evaluating TNF-α protein levels. Our data are certainly in agreement with the documented HO-1 activity in promoting the survival and cellular response against oxidative damage that is promoted by HG [22].

Our results support the idea that metoprolol counteracts the detrimental effects of hyperglycemia by coupling a direct mechanism, such as the selective β1-AR antagonist, that could participate in ERK1/2 activation in HG conditions. Moreover, metoprolol seemed to exert an intrinsic HO-1 inducer activity through the induction of the Keap1/Nrf2 axis.

Considering the aim of our study, we speculate that the down relation of Nrf2 repressor Keap1 could represent the main event that drives Nrf2 nuclear accumulation and the associated HO-1 up-regulation. These points will be better elucidated in future studies.

## 5. Conclusions

Our results confirm the pivotal role that is carried out by β1-AR in retinal angiogenesis, resulting in hyperglycemia. Inversely to β3-AR, β1-AR-mRNA levels significantly increased in HG-treated HREC. In the Matrigel tube formation assay, the inhibitory effect of metoprolol on the angiogenic potential in HG was significant as it succeeded in restoring the viability and tube formation capability, thus reducing HG-induced endothelial injury. The β1-AR blockade prevented HG-induced effects upon the metabolic activity of HREC. Metoprolol down-regulated the ERK1/2-cPLA_2_-COX2-PGE2 axis in HREC that was treated with HG and counteracted glucose-induced ROS accumulation by the eliciting the Keap1/Nrf2/HO-1 pathway. Moreover, we confirmed the increased production of IL-1β, TNF-β, and VEGF, and provided the first evidence that their release could be modulated by the β1-AR blocker during HG treatment of HREC. We understand that extrapolating these data to the in vivo condition is difficult. We are aware that a limitation of this study is the absence of in vivo data and that the role of β1-AR in hyperglycemic conditions will need to be further investigated with studies that are based also on in vivo experiments to develop new therapies aimed at counteracting diabetic retinopathy.

## Figures and Tables

**Table 1:** Primers sequences that were used for quantitative PCR.

Gene	Sequence (5′–3′)	Amplicon (bp)	Accession n.
VEGFA	Fw: ATCTTCAAGCCATCCTGTGTGC	121	NM_001025366.3
	Rv: GAGGTTTGATCCGCATAATCTG		
IL-1β	Fw: AGCTACGAATCTCCGACCAC	186	NM_000576.3
	Rv: CGTTATCCCATGTGTCGAAGAA		
TNF-α	Fw: AGCCCATGTTGTAGCAAA CC	134	NM_000594.4
	Rv: TGAGGTACAGGCCCTCTGAT		
18S rRNA	Fw: TAAGTCCCTGCCCTTTGTACACA	69	NR 146119
	Rv: GATCCGAGGGCCTCACTAAAC		

## Data Availability

The data presented in this study are available on request from the corresponding author. The data are not publicly available due to reasons of privacy.
